# Supplementary material for: Nanometer scale difference in myofilament lattice structure of muscle alters muscle function in a spatially explicit model
Source: PLoS Comput Biol. 2025 Apr 7;21(4):e1012862. doi: 10.1371/journal.pcbi.1012862 (PMC11975381; doi:10.1371/journal.pcbi.1012862)
Supplement: Attachment — Submitted filename: MuscleModel_plos_latex__2022___Rev_Response_.pdf [file pcbi.1012862.s002.pdf]

Nanometer scale difference in myofilament lattice structure of muscle alters muscle function in a spatially explicit model

Travis Tune[1,3] and Simon Sponberg[1,2]

[1]School of Physics, Georgia Institute of Technology, Atlanta, Georgia, USA
[2]School of Biological Sciences, Georgia Institute of Technology, Atlanta, Georgia, USA
[3]Center for Translational Muscle Research, University of Washington, Seattle, Washington, USA

Reviewer 1

This paper describes a model of a sarcomere that was deployed to demonstrate how a small change in myofilament structure (geometry) can have a profound impact on function including work produced. The main message of the paper in possibly explaining previous experimental observations is of wide interest to the field, however, the presentation of the paper makes it hard to access. The following are specific comments:

Major Comments

1. The introduction is a bit convoluted and longer than necessary. For example, the paragraph that starts on line 73 is already putting this manuscript into context, but then the paragraph on line 86 goes back into overall background. The introduction should end with a tight paragraph that clearly explains how the paper plans to address standing challenges in the field.

Thank you for prompting these changes. We have significantly revised the introduction. We tightened up several of the first paragraphs and made sure that the flow was consistently from big picture to more narrow question. We removed the paragraph in question moving essential points to the discussion. We add a little bit more about the why we vary lattice spacing as a parameter in the model to address Reviewer 2's core comment and a few other points in the reviewer. We now end with a specific outline and impacts paragraph.

2. Figure 5 would greatly benefit if there was something else to compare it to. As it is, the plots could be interpreted as being far away from experimental data (none of them match) or if other models are not able to replicate the qualitative behavior, this could be interpreted as a significant step forward. As it is, the reader is left floundering.

We have modified figure 1 to include the final work vs phase simulations (fig 5) after the modifications we made in the paper compared the both *M. sexta* and the previous model version's results. This better shows the 'before and after' of our modifications.

3. The methods portion is lengthy - the language could probably be tightened for a more efficient section.

We changed the model overveiw and geometry section to be more concise, since it is mostly explanation of the previous version of the model. Under the 'Rate Functions' section, we wanted to give a detailed list of the changes we made to the rate functions and crossbridge parameters, since they were fairly significant, and if future uses decide to modify the model further, we wanted to note all the changes we had made and why.

4. Overall, the presentation of the model and data is convoluted and difficult to follow due to typos, missing units, inconsistent terminology, lacking legends and captions, etc. Here are some examples and details:

   a. The authors are inconsistent with their use of the terms "axial spacing," "actin-myosin spacing," "radial spacing," and "lattice spacing." Initially "axial" is used to describe strain,

force, or overlap in the direction parallel to filaments and "radial" is used to define force or spacing between thick and thin filaments (Line 22-33; line 136-150; line 228). "Axial" is defined similarly with respect to "lattice spacing" later in lines 93-102 and Fig. 3. Figure 2 then defines the term "actin-myosin spacing" as it is mainly used later in the paper, much before it is discussed in the text (Section 2.6). At a minimum, Fig. 2 should be referenced in the discussion of actin-myosin spacing in the methods; Section 2.6 should be moved immediately after Section 2.2 to improve flow. The inconsistency lies at the start of Section 3.2, line 409 where the author refers to small differences in "axial spacing" of the myofilament lattice, where the term actin-myosin spacing should be used instead (referenced as lattice spacing later in paragraph). If the terms lattice spacing and actin-myosin spacing are indeed interchangeable, I recommend that only 1 be used for consistency throughout the paper (they are referenced separately in back-to-back sentences lines 424-425). This spacing is once again redefined as "filament separation" in Figure 8. The inconsistencies make the text very difficult to follow.

Thank you, the 'axial' term in line 409 was a typo and should have read 'radial.'

The terms 'lattice spacing' and d_{10} are synonymous and are experimentally measured via x-ray diffraction. The parameter we vary in the model is the radial distance between the thick and thin filaments, which is linearly related to the d_{10} spacing. We included the actual argument so that the simulations we did would be easier to replicate. The relationship is described in section 2.6. We moved that section to now be section 2.3.

b. Several figures reference panels by letters, but the figures do not include the corresponding labels (Fig. 5). Other figures reference panels that are inconsistent with the panel labels (Fig. 2A and B). Yet other parts of the text reference panels that do not exist (line 280)

We have removed the references to panel letters and amended the text accordingly.

c. The lattice shown in Fig. 2A should include a legend. The states should be labeled by number in Fig. 2C as well as the transition rates between them so that it ties to the references made in the text.

We have added a legend and added the transition rate labels to figure 2.

d. More on typos and grammar in minor comments below

5. The authors claim that increasing torsional stiffness decreases network in areas already associated with negative work is inconsistent with the yellow/green region in Fig. 7C. This discrepancy should at least be mentioned in the discussion.

We have amended the text of section '3.4 Cross bridge stiffness can attenuate or accentuate net work dependence on lattice spacing.'

6. The discussion of titin's effect on net work is minimal, and anticlimactic, especially given that the species this study is basing its simulations on does not actually possess titin. Further, the fact that myosin's torsional and linear springs were increased by factors of 10 and 4 respectively from 2sXB to match M. sexta cross-bridge tetanic forces, and later in vivo passive stiffness is mentioned to be much higher than in the model and to have a significant effect on work loops (Fig. 5), it follows that a change in titin stiffness (and perturbations about this change) may demonstrate greater effects on net work as seen in Fig. 5. In fact, titin's stiffness's potential effect on work is directly mentioned in line 398-399. If titin's stiffness is meant to be the concluding figure of this study, it warrants more investigation, otherwise, its discussion should occur earlier in the results section.

Insects, including *M. sexta*, contain elastic proteins such as sallimus, kettin, and projectin which perform the analogous function of titin in these animals [12]. The specific protein in cockroach muscles is unknown to the best of our knowledge. Because titin and titin-like proteins are thought to be important in the regulation of the lattice, we wanted to include simulations showing if changes in it had any effect on net work. We did not find any, but as noted in Figure 5, the passive forces in our model are far lower than in *M. sexta*, so the force of titin in the model is probably underestimated. We have added these details to the text.

7. The first paragraph of the discussion sections (505-514) was one of the best parts of the paper that really brought home the major contribution of this work to the field. However, the rest of the discussion section was hard to follow and overly long (ex. page 19 paragraph 541-558 could really be condensed to a couple of sentences).

Thank you for highlighting the first paragraph, we agree it is a good focal point for the paper. We have now revised the rest of the discussion to improve readability and organization. We now have one section discussing lattice spacing and the specific results of this study. We also incorporated responses to the other reviewer's comments here, reduced other paragraph, and have significantly shortened and revised the paragraph that was on lines 541-558). The second major section of the discussion, highlights the improvements to spatially explicit models in general and other factors in the application and interpretation of these models based on our later simulations.

8. There is a lot of discussion on radial force in both the introduction and discussion, but it didn't seem to be the main point of the paper.

In our model, radial forces have no direct effect on the results, but in real muscle the radial force generated by crossbridges is the primary factor responsible for increasing or decreasing the lattice spacing. Because changing the lattice spacing can either increase or decrease further crossbridge binding, the radial forces are important potential indicators of the lattice dynamics. See further response to Reviewer 2's first comment.

Minor Comments

1. M. Sexta experimental data is referenced in several captions, citations should be made available in the caption as opposed to having the reader return to the main text to find the references (3 mentioned initially in intro [26, 28, 29], only 1 mentioned elsewhere [28]).

We have added citations everywhere previously published *M. sexta* data is used. The twitch and tetanus data show in Fig. 4 and section '2.5: Activation profile was found by matching to twitch force' were collected by us for this publication.

2. How axial separation is calculated in Fig. 3 is unclear, the caption should include a clear definition. Additionally, the units for actin-myosin spacing are missing in the caption.

Axial separation is the axial distance between the base of the myosin head (the nodes in the model diagram figure) and the nearest actin binding site. We have added this definition to the rate function figure, and indicated it in the model diagram figure part B.

3. Why is Fig. 7A green dotted while a solid line in Fig. 7B. Mention that the in vivo lines are green in the caption to be consistent with the prior two conditions. L0 in the caption should be LS0 to match the axis in 7B.

We have changed the green lines to be dotted and amended the caption.

4. Whether lattice spacing is constant or dynamic during simulations should be clearly mentioned in captions (e.g. Fig. 6 and Fig. 9)

We have clarified in the captions that the lattice spacings there are constant.

a. Units in Fig. 6 caption

We have added units to the caption.

b. Fig. 9 caption: "The condition b = 4 um-1 is the condition used in all simulations above" appears to refer to the figure, not prior simulations. This standard titin stiffness should be mentioned in the methods where Ftitin is initially defined.

We have added the parameters describing the force of Titin to table 1 "Model Spring Parameters".

5. Phases mentioned in line 448 include significant figures not mentioned in Fig. 7A labels.

We have corrected the phases to indicate what was simulated, and what is the in vivo limits of M sexta

6. There are quite a few grammatical mistakes in the paper: a. Lines 9-10 (page 2), sentence fragment – not clear what is meant.

Thank you, we have fixed the typo.

7. There is also writing that is hard to follow as there are missing logical linguistic links to guide the reader. For example, the last sentence of the paragraph on line 32-33 (page 2) needs to be logically linked to the rest of the paragraph.

Thank you, we have revised the introduction.

8. Missing citations on Line 323-325 and Line 376-377.

We recorded the passive and active twitch force ourselves following an experimental protocol similar to that of [10]. We have added this to the text under section '2.5: Activation profile was found by matching to twitch force.'

In summary, this work has the potential to be of interest to the field, but the manuscript would need to be significantly re-written prior to publication.

Reviewer 2

The authors demonstrate importance of spatially explicit muscle modeling and namely effect of distance in actin-myosin lattice. They adjusted the model from prior works by comparing the stress to a flying muscle of a moth. On the model, with work output profiles reminding those of model organism, the found, that first, any difference in (constant) lattice spacing (LS) greatly affects the muscle work output and second, the physiological time-varying lattice distance result in almost same net work as constant LS.

This seems to me it is more of a collateral effect than a driving principle. As such, it needs to be described, but its effect on simulations is probably low. Physiologically, if the lattice was observed to be different, then the spring lengths, stiffnesses and default angles would have also been different. Although this is acknowledged in discussion on line 528, this chicken-egg problem is substantial caveat of the paper and the imposed differences of the distance should be considered as hypothetical.

There might be an opposite use case for the model though - what is the optimal spacing given the chosen set of k and r? And, alternatively, what is the otpimal k and r, given the observed lattice spacing, which can be better measured? That said, although the developed model has a wide demonstrative potential and it is tempting to show all of its capabilities, I recommend the authors to focus on less.

The lattice spacing can be affected by multiple factors. Besides the radial force generated by crossbridge activation, there are other regulatory proteins which also apply radial forces, and therefore contribute to the overall lattice spacing. For example, in rats with Duchene Muscular Dystrophy (DMD) the protein dystrophin is absent, which was observed to cause an increase in the average lattice spacing, and lower crossbridge recruitment due to the actin filaments being further away radially [6]. This is because dystrophin sits inside the sarcolemma and can be thought of as applying an inward radial force on the myofilament lattice, making it an important regulatory protein [4]. There are also other regulatory proteins in the z-disk and throughout the lattice, some of which may even be calcium sensitive. So it is not the case that the spring properties of the crossbridges would have to be different between two hypothetical muscles in order to have different lattice spacing dynamics. Changes in other regulatory proteins would be sufficient.

Hypothetically, one could imagine having a model which applies a radial force to the lattice which models radial forces from regulatory proteins. In that case, the lattice spacing would be emergent from the balance of radial forces due the crossbridge heads (modeled here by the two-spring system), and the model chosen to represent the net effect of other radial forces. Titin, for example, is believed to have a role to play in the regulation of the lattice spacing, though since titin may be calcium sensitive, such a model may be quite complex. A simple model of dystrophin might be a linear spring.

However, doing this is very difficult computationally. At each timestep, the lattice spacing would have to be found by balancing all the different contributions of radial force so that the net force is zero. Because the system is nonlinear, it can not be solved directly. Furthermore, we already must balance all forces on the nodes of the thick and thin filaments in the axial direction, and changes in the radial configuration would affect the axial forces as well. Even for a very simple model of the external radial forces, the overall system would be highly nonlinear, and it would be very difficult computationally to balance the radial and axial forces simultaneously.

For the above reasons, we instead change lattice spacing directly. This can be thought of as being equivalent to assuming a model for the (non-crossbridge derived) radial force which happens to result in the lattice spacing prescribed. While it could be argued that the assumed radial force model may or may not be physiologically relevant, by including lattice spacing changes measured from *M. sexta*, we can ensure the trajectory of lattice spacing changes (which is due to the balance of all radial forces) is derived from physiological data.

We have added details to section '4.1: Lattice spacing and crossbridge stiffness mediate multiscale interactions that alter whole muscle function.'

Major Comments

1. An issue I was often facing throughout the paper is that the potential reader might not be familiar with the prior works, yet should still be able to understand the presented work. Namely, please explain in more detail the work loop at different activation phases and / or provide an explanatory figure, incl. sarcomere lengths. Is it that you shifted strain and activation? Can you explain why the maximal power output is at 0.8, while the physiological in vivo activation phase at 0 (line 419) is suboptimal?

The workloop is a technique in which the muscle is subjected to a strain amplitude, oscillation frequency, and pattern of stimulation similar to what it might experience under *in vivo* conditions, in contrast to isometric, isotonic, or constant calcium concentration. Strain amplitude, frequency, and timing/phase of activation can be measured though techniques such as high speed video or tethered locomotion simultaneous with recording electrical activity of the muscle. The phase of activation is the phase relative to the length cycle at which stimulation occurs, and can be shifted relative to strain. It is intended to examine muscle's behavior under a range of conditions which more resemble the physiological state of the muscle during locomotion. In tethered flight in *M sexta*, phases of activation have been observed from between 0.8 (max power) and 0.2. The average *in vivo* phase of activation (0, start of shortening) occurs at a point in the power-phase curve where the slope is steepest, meaning small changes in the timing of activation have a large effect on power output. One explanation is that this gives a large amount of control to the organism, allowing it to modulate power output for controlled flight [9].

2. Similarly, the modified model needs to be described in a little more detail, to have a basic understanding without the need of reading the prior works first. Namely the figure 2 can use some refinement to make it more illustrative.

a. Figure 2A: The figure suggests you are calculating interaction of a single myosin and 6 actins - is this correct? What role do the additional 2 actins and three myosins play then?

We did not mean to imply only those myosin and actin were interacting. The model contains 4 myosin and 8 actin, and each myosin interacts with 6 actin. This is done through periodic boundary conditions, with the bold filaments indicating the actual filaments, and shaded filaments indicating pseudo-filaments which are actually carried out by interactions with a filament on the opposite side. We have modified the figure to better show this.

b. figure 2B - if the crowns are in triplets, the bottom- and top-facing heads should probably alternate down, top, down, top... correct?

The crowns rotation is 60°, 60°, 0°, as found in [1], so the orientation should be down, top, down, down, and we have changed the figure to reflect that. For clarity we have also only shown a few of the 720 crossbridges.

c. Rates are not labeled in fig 2C - label the transition and rates accordingly.

We have added the rate labels to the diagram.

d. Please be explicit about the activation function - what parameters were tuned in the current work?

We have added the actin permissiveness function to the paper, at the end of Section 2.4, 'Actin permissiveness parameterizes Ca2+ and tropomyosin dynamics.'

e. Please explain the role of k and r in generating the force. I assume it follows F ∼ (x-r)*k. But then the r of the thick and k of the thin filament have strange units and R of the thin filament is not defined (probably 0?)

The force of a crossbridge is analogous to F∼ (x-r)*k, but because of the torsional spring it is given by $F(r, \theta) = k_r \Delta r_{w,s} + k_\theta \Delta\theta_{w,s}$, with w, s indicating the weak or strongly bound state, and with r and θ being the polar representation of the axial separation x and the actin-myosin separation (LS), with LS being the primary parameter we vary in this paper. The units are because the stiffnesses and free energies are given in units of KT.

f. Line 290: How does setting these parameters arbitrarily align with previous justification of the values?

The previous average force of a crossbridge was low compared to experimental results [7] using the previous spring stiffnesses values, so we increased them to be in line with physiological data. We have amended the text.

g. Figure 6 caption: The stochasticity is not due to the model being spatially explicit, but the noise is high because the model is stochastic. Can the noise by tuned down with higher number of averaged periods? It is not common to see simulation results being more noisy than

the measurement. Prior description of the model in the current paper does not make obvious whether the model is deterministic or not.

Because the model is spatially explicit, the state of every crossbridge and the force they produce is tracked individually and state changes are random, according to the rate functions. The noise is due to the fact that we only simulate 4 thick and 8 thin filaments with 720 crossbridges total, of which only about 10% are active during peak contraction. So because the force is composed of a small number of crossbridges, each of which produces a different amount of force, there can be a significant amount of noise. Therefore we simulate 20 periods and average them together to generate the work loop plots shown. Further averages could improve the signal to noise ratio, but come with computational cost.

h. Line 346: please explain the relation of spacing to d10 - does it mean the radii of actin and/or myosin in vertebrate muscle is much smaller? Similarly line 412 - how the spacing of -1nm makes lattice -1.7nm?

The d_{10} is measured by x-ray diffraction and is the distance between diffraction planes composed of myosin, as shown in Fig. 2A. The spacing between different filaments depends on the geometry of how actin is distributed throughout the myosin lattice. The spacing we change in this model is the Myosin-Actin spacing, however, the relationship will depend on the packing geometry of actin relative to myosin. For example in vertebrate muscle, and the work presented here, the actin filaments are located centered between three myosin filaments (as in Fig. 2A), whereas in invertebrate flight muscle, the actin filaments are located between two myosin filament ([5], fig 15A). Invertebrate leg muscles can have yet another packing geometry, and have double the number of actin filaments relative to invertebrate flight muscle ([5] Fig. 15B). We have added the conversion between d_{10} and the model parameter LS in the text of section titled 'Actin-Myosin Spacing and d_{10}.'

3. Section 3.1: Consider moving the protocol description to the methods and concentrating on the true results here.

We moved the description of the work loop protocol to the end of the methods.

4. Further sections (3.4 and 3.5) are a bit confusing as of the point the authors are trying to make. Static parallel stiffness of titin should not affect the work in principle (or should it?). The conclusion on line 502 is not supported by these obsrvations - the model simulation simply did not reproduced this phenomenon, if it was present at all. Looking aside from the net work, do these experiments show difference in total force, contraction speeds or some other important factors of the muscle contraction?

Previously, in other versions of the model, it was seen that changing the stiffness of titin altered the rate of crossbridge cycling and how much isometric force could be generated [8]. This is likely due to the spatially explicit nature of the model. The compliance of the filaments, including titin, affects how much crossbridges move axially due to the force of neighboring crossbridges [3]. We wanted to include simulations showing changes in titin's stiffness since it is thought to be a big contributor to the structure of the lattice spacing. However, we only simulated sarcomeres of 2.5 µm, and in [8], titin only had a substantial effect at larger sarcomere lengths. We have included these details in the text.

Minor Comments

5. Line 117 - the 2sXB is not defined.

We appologise for not including it. The 2sXB model was introduced in [11]. It was termed 2sXB to indicate the torsional 2 spring system which makes up the crossbridge head, as opposed to a single linear spring in the axial direction. We have indicated this in the text, in the second paragraph of the subsection 'Model Overview.'

6. Model geometry - I do not understand why you chose such subset - there are only 6 active connections, as in 2A? Then the myosin heads to actin ratio should be 6:1 in 2B?

2A shows a cross-sectional view of the sarcomere, while 2B shows a longitudinal view. Since the structure is 3D, we can't show every filament. In 2B, for clarity, we only show a single thick and thin filament. However, there will be 6 thin filaments in total for each thick filament.

7. What is the poisson's ratio?

Poisson's ratio is the ratio of strain changes in the lateral (radial) direction compared to the strain changes in the axial direction: $\nu = \frac{d_{radial}}{d_{axial}}$. For an isovolumetric compression, the ratio should be 0.5.

In the case of a constant lattice spacing, Poisson's ratio is 0. While muscle is isovolumetric on longer timescales, during high frequency phasically activated stress strain cycles like work loops, the lattice is not necessarily isovolumetric. Because of the radial forces produced by crossbridges Poisson's ratio for muscle can even be negative, indicating that axial compression results in radial compression. We have included the definition in the last paragraph of the Intro.

8. What is a protocol for fig 1? At which SL?

   The experimental data presented was from whole muscle (dorsal longitudinal muscle, DLM) from *M. sexta*, first published in [10]. The simulated data was done at a sarcomere length of 2.5 µm.

9. typo 231 and 262: 2xSB

   Fixed

10. line 295 - did you mean pN?

    Fixed

11. Line 339: typo piror

    Fixed

Reviewer 3

The manuscript by Tune and Sponberg extends a set of spatially-explicit models integrating radial and axial forces in the cross-bridge binding activity to explore the effects of muscle contraction during a simulated workloop. The authors compare radial compression of the myofilament lattice and stiffness values of the cross-bridge to show that changes in spacing and stiffness can augment or diminish the amount of work produced by the muscle. Authors compare these data to experimental data from Manduca Sexta, albeit somewhat qualitiatively. Clearly there are quantified numbers showing the differences between positive work production vs. negative work absorption, but it is difficult to compare the numbers directly. The main take away from the computationa simulations is the authors trying to show that lattice spacing is very important for dynamic contractility, work, and power production. This is an interesting idea.

Detailed comments are listed below:

Major comments

Methods, section 2.1, page 4. It is a little unclear if the model being used or discussed solely accounts for axial force balance and force generation from time-step to time-step vs. axial and radial components of the calculations. From Fig. 2 it is clear there are axial and radial aspects of the cross-bridge properties, but it seems these axial and radial cross-bridge calculations do not translate into the axial force balances utilized to calculate the force produced during the simulation. Please clarify or advise.

In the model each crossbridge does generate both radial and axial forces, due to rotation about the torsional spring. The axial component of this force is used in force balance along the axial direction. However, the radial force is not used in any kind of radial force balance. This is because we would require a restoring force to balance against which would be due to the various anchoring proteins in the z disk and m-line, and we don't have a model for those forces. We can however measure the amount of radial force generated by crossbridges in the model, and we also know from experiments that that force is of a magnitude which can affect the lattice spacing, either increasing or decreasing it [2]. To get around the lack of the radial force balance in the model we prescribe the lattice spacing using the d_{10} measured in *M. sexta* via x-ray diffraction, which is our attempt to account for the feedback between crossbridge binding and lattice spacing. We have added details regarding the force balancing and added the equation for force into the Section titled 'Model Geometry.'

Methods, Table 1. Characteristics or details related to the Thin Filament are not clear or may be missing proper units. Perhaps description of the table or a legend could be helpful.

We have revised the methods section so that every equation or value appears next the text describing them.

Methods, page7, line 167: "Myosin head triplets are azimuthally distributed by 120° and adjacent crowns are rotated by 60°." Please clarify or advise. Is this packing supposed to represent the insect flight muscles that are being simulated? I don't know the insect flight muscle packing exactly, but this could be clarified or referenced. I may be incorrect, but this packing is not consistent with vertebrate

cardiac, nor skeletal, muscle packing of the myosin crowns. Specifically, I think the degrees of rotation per crown appears incorrect.

We apologize for the confusion. In this model subsequent myosin crowns are separated by angles of 60°, 60°, and 0 degrees, which was found in [1] in rabbit cardiac muscle. They termed this a 'perturbed' crown orientation, compared the an 'unperturbed' crown angle of 40°. We have corrected the text.

Methods, page 7, line 176. In discussing or listing the stiffness values for the thick and thin filaments—these are all from vertebrate skeletal muscle measurements (I think as authors also reference). How might this compare to what is known about the insect muscle stiffness values the authors are trying to simulate or emulate with their workloops? For example, aren't the stiffness characteristics of fruit fly and lethocerus muscles much stiffer than vertebrate muscles? Have the authors considered the implications of this for their simulations or the interpretation of the data in their study? Have the authors considered a set of simulations with much stiffer thick filaments and much floppier thick filaments, for example, to compare with the current simulations?

In the original version of this model [3] they considered the effect of changing compliance in thick and thin filaments. Because myosin motors are elastically linked, they saw that axial forces in the crossbridges were inhomogeneous, which resulted in realignment of binding sites in response to crossbridge forces. Due to this realignment, a kind of coupling can be observed, in which crossbridges which initially are too far away *axially* from a binding site can be dragged close enough to bind. That model's crossbridges included axial forces only, whereas here we were interested in examining an analogous effect, except in the radial direction instead of axial. We didn't want to change the filament stiffness, since it does not generate a force in the radial direction. However, since in [3], they found that changes in filament stiffness could increase or decrease total isometric force and crossbridge turnover, and since crossbridges generate a radial force, we might expect axial stiffness to have an indirect effect on the total radial force generated in muscle. We have added details about this in the section on 'Model Geometry.'

Methods, Page 11, Section 2.4 and Fig. 4B. The concept of actin permissiveness is very unclear. Is this some probability of binding for the whole system or for a single actin binding site etc. What gives the permissiveness a temporal dynamic that rises and then falls, or where is the mathematics driving tis behavior? How is this dealt with related to spatial components of activation vs. the probability of activation at any single binding site vs. activation of the system as a whole?

The actin permissiveness is a factor from 0 to 1 which represents the average state of actin binding site availability. It is essentially a number which is intended to capture the dynamics of Ca^{2+} influx, tropomyosin binding and conformational changes, and outflow. A value of 0 indicates Ca^{2+} concentration is too low to cause any of the actin binding sites to be unblocked, while 1 indicates Ca^{2+} concentration is high enough that all binding sites are accessible. Computationally, we first calculate the binding probability due to geometry alone based on the axial and radial separation between the head and binding site, for each head-binding site pair. That geometric probability is then multiplied by the actin permissiveness to get the total binding probability. The actin permissiveness is a global value. We have clarified the actin permissiveness in the section titled 'Actin permissiveness parameterizes Ca^{2+} and tropomyosin dynamics'

Results, Page 12, Section 3.1. Please remind or clarify the definition of the phase of activation timing. When it is related to which aspects of the wingbeat or the shortening and lengthening of the muscle. As in where is phase =0 and phase =0.5. I realize you define this related to Fig. 5 and the legend of Fig. 5, but something seems confusing about these assumptions or their implementations.

We define a phase of 0 as the start of shortening, which is equivalent to the beginning of the downstroke of the dorsal longitudinal muscles (DLM) in the wings of *M sexta*. We have clarified this in the second paragraph of the results.

Results, pages 12-13. Is there any phase shift or viscous delay of the passive component of the workloop? Did the authors simulate the passive workloops to subtract them from the activated workloops? Since the model is purely elastic, likely not any temporal shifts or phase delays. Please comment on details or show the passive simulations if they are interesting and important to illustrate important aspects of these assumptions for subtracting the passive components.

In our simulated workloops the passive force is purely elastic and there is no viscous component to the passive workloop. In real muscle though, there may be some passive element. We chose to subtract the passive component of the force for the Manduca workloops because it is much larger than the passive force present in our simulations, relative to the peak active force.

Results: It is a neat idea and a nice set of simulations showing that lattice spacing changes can amplify or dissipate the net work throughout a dynamic contraction of a workloop. However, it is not clear why this occurs. Is it really as simple as the cross-bridge stiffness? How does this sensitivity to the cross-bridge stiffness scale or become affected by the kinetic rate parameters chosen to underly the cross-bridge binding and cycling? Have the authors considered a sensitivity analysis to further investigate these details for which portions of the chemomechanical cycle this finding stems from?

The lattice spacing is effectively the radial distance that a crossbridge must traverse in order to bind to an actin binding site. If the distance is too great, binding can't occur. For smaller distances, binding probability will increase to some maximum. In addition, the decomposition of force into both axial and radial directions will change with lattice spacing. Therefore, because of compliance in the radial direction (which was not explicitly modeled in this work, but is present in real muscle), crossbridge binding and the lattice spacing will be coupled together, similar to how axial spacing and crossbridge binding were found to be coupled in [3]. The stiffness of the crossbridge head is one factor which can effect these phenomena, since it effects both the force produced, the share of force in the axial and radial directions, and the kinetics themselves (since the state transitions are based on the free energy, which is bases on the spring potential energy). Because there are so many parameters, it is difficult to do a sensitivity analysis on more than a few parameters, and we believed that the crossbridge stiffness combined with lattice spacing changes would have the most direct effect on net work produced.

Minor Comments

Results, page 15, line 462. Missing units on the lattice spacing value(s). Fixed

Discussion, page 19, line 542. Work in this sentence seems to be missing units. Fixed

Discussion, page 21, line 619. Interesting ideas about the thick to thin filament ratios. There are also differences in the crown packing or myosin crown organization among taxa that would be helpful to comment or consider here too. Thank you for pointing this out. We have added a comment about myosin crown structure the the text.

References

[1] Hind A. AL-Khayat, Edward P. Morris, Robert W. Kensler, and John M. Squire. Myosin filament 3d structure in mammalian cardiac muscle. *Journal of Structural Biology*, 163(2):117–126, 2008.

[2] G Cecchi, MA Bagni, PJ Griffiths, CC Ashley, and Y Maeda. Detection of radial crossbridge force by lattice spacing changes in intact single muscle fibers. *Science*, 250:1409–1411, December 1990.

[3] Thomas L Daniel, Alan C. Trimble, and P. Bryant Chase. Compliant realignment of binding sites in muscle: Transient behavior and mechanical tuning. *Biophysical Journal*, 74, 1998.

[4] Q. Q. Gao and E. M. McNally. The Dystrophin Complex: Structure, Function, and Implications for Therapy. *Compr Physiol*, 5(3):1223–1239, Jul 2015.

[5] B. Millman. The filament lattice of striated muscle. *Physiological Reviews*, 78:359–391, apr 1998.

[6] Saffie Mohran, Chen-Ching Yuan, Shawn M. Luttrell, Weikang Ma, Thomas C. Irving, David L. Mack, and Michael Regnier. Development of mechanical and structural dysfunction in skeletal muscle from a duchene muscular dystrophy rat model. *Biophysical Journal*, 118(3):277a–278a, Feb 2020.

[7] Gabriella Piazzesi, Lenoardo Lucii, and Vincenzo Lombardi. The size and the speed of the working stroke of muscle myosin and its dependence on force. *The Physiological Society*, 545:145–151, 2002.

[8] Joseph D. Powers, C. David Williams, Michael Regnier, and Thomas L. Daniel. A spatially explicit model shows how titin stiffness modulates muscle mechanics and energetics. *INTEGRATIVE AND COMPARATIVE BIOLOGY*, 58(2):186–193, AUG 2018.

[9] S. Sponberg and T. L. Daniel. Abdicating power for control: a precision timing strategy to modulate function of flight power muscles. *Proceedings of the Royal Society B: Biological Sciences*, 270:3958–3966, July 2012.

[10] Michael S. Tu and Thomas L. Daniel. Submaximal power output from the dorsolongitudinal flight muscles of the hawkmot manduca sexta. *Journal of Experimental Biology*, 207(26):4651–4662, 2004.

[11] C D Williams, M Regnier, and T L Daniel. Axial and Radial Forces of Crossbridges Depend on Lattice Spacing. *PLOS Computational Biology*, 6(e1001018):1–10, December 2010.

[12] Chen-Ching Yuan, Weikang Ma, Peter Schemmel, Yu-Shu Cheng, Jiangmin Liu, George Tsaprailis, Samuel Feldman, Agnes Ayme Southgate, and Thomas C. Irving. Elastic proteins in the flight muscle of manduca sexta. *Archives of Biochemistry and Biophysics*, 568:16 – 27, 2015.
