# Supplementary material for: Nanometer scale difference in myofilament lattice structure of muscle alters muscle function in a spatially explicit model
Source: PLoS Comput Biol. 2025 Apr 7;21(4):e1012862. doi: 10.1371/journal.pcbi.1012862 (PMC11975381; doi:10.1371/journal.pcbi.1012862)
Supplement: Attachment — Submitted filename: MuscleModel_plos_latex__2022___Rev_Response_Round_2_.pdf [file pcbi.1012862.s003.pdf]

Nanometer scale difference in myofilament lattice structure of muscle alters muscle function in a spatially explicit model

Travis Tune[1,3] and Simon Sponberg[1,2]

[1]School of Physics, Georgia Institute of Technology, Atlanta, Georgia, USA
[2]School of Biological Sciences, Georgia Institute of Technology, Atlanta, Georgia, USA
[3]Center for Translational Muscle Research, University of Washington, Seattle, Washington, USA

Reviewer 1

The authors have addressed most of my comments. A few minor issues remain:

1. Figure 1 - the way the arrows are set for the inset, it was very hard to figure out what was going on. Additionally the colors are very confusing where it seems that green means one thing in one panel and another in the other panel. The left panel should have a box that indicates what the inset is zooming onto.

We are sorry for the confusion. The green lines in the two plots are the same data. We have revised the plots to better indicate that the right plot is an inset of the left.

2. Figure 2 - the legend inside the figure should also cover the symbols in the axial view and rate diagram.

Thank you, we have added the other items to the legend.

Otherwise, I believe the paper will be of interest to the readers in this field, and the manuscript was much easier to read with the new revisions.

Thank you for time and effort in reviewing the paper.

Reviewer 2

I want to congratulate the authors on substantially improving the paper. The aims are much clearer and the methods section are better understandable now. Still, I have found some minor issues.

The thick and thin filament parameters presented after line 163 are confusing. What is a and b here and why do the thick and thin filaments have different units? Are the labels flipped and k_thick and r_thick should be used instead?

We apologize, the a and b were typos, and the table entries were transposed. We have corrected the table.

Section 3.5 I do not see, how the passive properties are related to the muscle work output. The titin is modeled without hysteresis so it may not affect the work per se. Moreover, as admitted, the modeled passive force is minor compared to the one in vivo. Although this observation might be really interesting, it lacks any experimental support as well as mechanism in the model. I suggest dropping this whole section, noting in the end of section 3.4 that the change in simulated net work was not observed for a change in titin stiffness for the reasons mentioned above.

The stiffness of titin can indirectly influence force because of the elastic properties of the thick filaments and the spatially explicit nature of the model. When a myosin head binds to an actin binding site, all other myosin heads and actin binding sites will experience a force, which results in myosin heads and actin binding sites moving relative to each other, either closer or farther apart, altering further binding probabilities. This was first reporting in [1], and was called ‘compliant realignment.’ In a later version of the same model, it was also been shown that the stiffness of titin could affect the isometric force, since the realignment of the crossbridges could be more or less depending of the relative stiffness of titin [2], since titin is in series with the thick filament. Because isometric force had

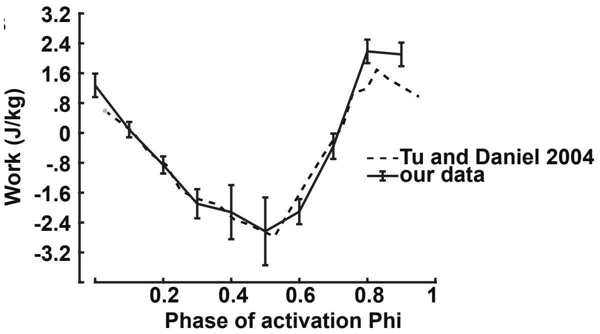

Figure 1

been shown to be affected by titin stiffness in the model in that work, done using the same model, we also wanted to see how work was affected. We have modified section 3.5 to better explain our reason for including that section.

LIne 378: How did you subtract the passive force from fig 5? I have not found any explanation in the original work [27].

We apologize for overlooking our explanation here. We used a nearly identical experimental rig and protocol and measured our own work loops under the same conditions as well as passive work loops. To make sure we were getting similar results, we compared our active workloops with those from [3] by plotting phase vs net work (Fig. 1). So the passive workloop we performed ourselves, but the active work loop data came from [3]. We have now mentioned this in the caption of Fig. 5.

Regrettably, I was not able to run the code due to security limitations of my current computer. The repository seems to be more than 3 years without any update, please check the source code is up-to-date with the plots in the paper.

The repository listed is the one which was used in the all simulations in this paper. The latest version, which includes a more complicated rate scheme for both the myosin heads and actin binding sites, is available at `https://github.com/travistune3/multifil_five_state`. This paper was written before the new version was available. The newer version was written in order to harmonize with experimental efforts aimed at affecting certain transitions in the crossbridge cycle at a finer level than the version used in this paper. We performed a few exploratory simulations using the newer version of the model to compare to the results of this paper and decided the results were not significant enough to warrant redoing all the simulations in this paper.

Fig 9 caption typo “three different vales”.

Thank you, we have corrected the typo.

LIne 300 The acronym DLM has been explained in a dropped paragraph. Please explain in text.

Thank you, we have added an explanation to the last paragraph of the introduction.

Reviewer 3

The paper is much improved.

With regard to the activation levels discussed in Fig. 4. Is the small activation level (i.e. maximal at 0.2 permissive value) in Fig. 4, right hand panel really actually used for all simulations? Thus, it is confusing about what happens with the tetanic stress response on Fig. 4 left side panel and what activation looks like related to that simulation response.

We apologize for the confusion. In tetanic simulations the activation level is held at 1. In passive simulations it is set to 0. In all other work loop simulations it is time varying according to what is shown in Fig. 4. We have clarified the caption and the section titled ‘Actin permissiveness parameterizes Ca^{2+} and tropomyosin dynamics’.

References

[1] Thomas L Daniel, Alan C. Trimble, and P. Bryant Chase. Compliant realignment of binding sites in muscle: Transient behavior and mechanical tuning. *Biophysical Journal*, 74, 1998.

[2] Joseph D. Powers, C. David Williams, Michael Regnier, and Thomas L. Daniel. A spatially explicit model shows how titin stiffness modulates muscle mechanics and energetics. *INTEGRATIVE AND COMPARATIVE BIOLOGY*, 58(2):186–193, AUG 2018.

[3] Michael S. Tu and Thomas L. Daniel. Submaximal power output from the dorsolongitudinal flight muscles of the hawkmot manduca sexta. *Journal of Experimental Biology*, 207(26):4651–4662, 2004.
